# Intravascular Imaging-Guided Versus Angiography-Guided Percutaneous Coronary Intervention: A Systematic Review and Meta-Analysis of Randomized Controlled Trials

**DOI:** 10.3390/diagnostics15091175

**Published:** 2025-05-06

**Authors:** Akash Kumar, Muhammad Salman Nadeem, Sooraj Kumar, Muzamil Akhtar, Ayesha Maryam, Rubyisha Sheikh, Nomesh Kumar, Naresh Kumar Ladhwani, Nimurta Madhwani, Nisha Kumari, Muhammad Riyyan Rao, Syed Sarmad Javaid, Peter Collins, Raheel Ahmed

**Affiliations:** 1 Medical Research Center, Liaquat University of Medical and Health Sciences, Jamshoro 76060, Pakistan; akashdeewan4@gmail.com (A.K.); kumarinimurta@gmail.com (N.M.); riyyanrao0@gmail.com (M.R.R.); 2 Department of Medicine, Nishtar Medical College, Nishtar Medical University, Multan 66000, Pakistan; m.salmannadeem736@gmail.com; 3 Department of Internal Medicine, Brookdale Hospital and Medical Center, Brooklyn, NY 11212, USA; sooraj_kumar94@hotmail.com; 4 Department of Medicine, Gujranwala Medical College, Gujranwala 52250, Pakistan; muzamilb112@gmail.com (M.A.); ayeshamaryam090@gmail.com (A.M.); 5 Department of Medicine, Karachi Medical and Dental College, Karachi 74700, Pakistan; rubyisha.sheikh@gmail.com; 6 Department of Internal Medicine, Detroit Medical Center/Wayne State University, Detroit, MI 48202, USA; 7 Department of Medicine, Dow University of Health Sciences, Karachi 74200, Pakistan; nareshkumarladhwani@gmail.com (N.K.L.);; 8 Department of Medicine, University of Mississippi Medical Center, Jackson, MS 39216, USA; sarmadjavaid1999@gmail.com; 9 National Heart and Lung Institute, Imperial College, London SW7 2AZ, UK; peter.collins@imperial.ac.uk

**Keywords:** percutaneous coronary intervention, intravascular ultrasonography, intravascular imaging, angiography, meta-analysis

## Abstract

**Background/Objectives:** Despite the potential benefits, intravascular imaging for guiding percutaneous coronary intervention (PCI) remains underutilized. Recent trials have provided new data, prompting a need for updated insights. This study aimed to perform a comprehensive meta-analysis to compare the clinical outcomes of intravascular imaging-guided PCI versus angiography-guided PCI, thereby evaluating the relative effectiveness of these two guidance strategies in improving patient outcomes. **Methods:** PubMed, Cochrane Library, Embase and Clinicaltrials.gov databases were systematically searched from inception till 25 November 2024. Randomized clinical trials (RCTs) comparing intravascular imaging with coronary angiography in patients undergoing complex PCI were included. Statistical analysis was conducted using a random effects model to calculate pooled risk ratios with 95% confidence intervals (CI). **Results:** In this meta-analysis of 21 studies involving 18,043 patients, intravascular image-guided PCI significantly reduced the risk of all-cause mortality by 24%, cardiac mortality by 63%, MACE by 35%, target vessel myocardial infarction by 32%, stent thrombosis by 42%, target vessel revascularization by 45%, target lesion revascularization by 34% and myocardial infarction by 22% compared to angiography-guided PCI. There was no significant difference in bleeding events. **Conclusions:** Intravascular imaging significantly reduces cardiac events, all-cause mortality and revascularization rates in PCI patients. These findings support its broader adoption and potential updates to clinical guidelines.

## 1. Introduction

Ever since it was introduced, coronary angiography has been the gold standard for diagnosing coronary artery disease and guiding percutaneous coronary interventions (PCI). However, it is innately limited in its functionality as it only provides a two-dimensional view of the blood vessels. Keeping in view this limitation, scientists have developed novel techniques such as intravascular imaging (IVI) which provides a three-dimensional view and not only aids in the better assessment of the plaque characteristics but also helps in a refined evaluation of the luminal stenosis [1]. This consequently results in better positioning of the stent and implantation of larger stent with increased minimal stent area. Some tools used in IVI are intravascular ultrasound (IVUS) and optical coherence tomography (OCT). These IVI techniques offer an optimized treatment which translates to improved outcomes in randomized controlled trials, namely: reduced major adverse cardiac events (MACE), target vessel failure and target vessel revascularization [1,2,3].

Despite the superiority of IVI over angiography for PCI shown by multiple trials and meta-analyses, it is not widely adopted clinically, with estimates reflecting usage in <15% of PCI performed [4,5]. This skepticism towards IVI can be ascribed to lack of acquaintance with the imaging equipment, gap in the knowledge for interpreting images, inadequate financial incentives and lack of comprehensive research data. Additionally, some clinicians are apprehensive about the perceived increase in procedural time, which they believe could negatively impact workflow efficiency [6,7]. Nevertheless, these concerns must be addressed.

The previous meta-analysis included 16 RCTs with a sample size of 7814 [2]. Since then, new RCTs have been published with more sample sizes. These recent studies provide valuable data and warrant an updated meta-analysis for synthesis of a more solid evidence and fill the gap in the literature. This new meta-analysis can help counter the cynicism that exists with regards to the adoption of IVI and can eventually improve patient outcomes.

## 2. Methods

This meta-analysis adhered to the Preferred Reporting Items for Systematic Reviews and Meta-Analysis (PRISMA) [8] and was prospectively registered on the international prospective register of systematic reviews (PROSPERO) (CRD42024605908).

### 2.1. Literature Search

A systematic search of PubMed, Embase, Cochrane Library and Clinicaltrials.gov from inception until 15 February 2025 for RCTs comparing IVUS or OCT guided PCI with angiography guided PCI was performed by two independent reviewers using a predefined search strategy. The details of the search strategy are provided in Supplementary Files. Acquired articles were then transferred to Rayyan.ai for full text screening by 3 independent reviewers for the final inclusion based on the inclusion and exclusion criteria. Any disagreements and discrepancies were solved by discussion with the senior author.

### 2.2. Eligibility Criteria

We included only RCTs that compared intravascular imaging-guided PCI with angiography-guided PCI. Eligible studies compared IVUS-guided or OCT-guided PCI either separately or in combination, with angiography alone, and reported at least one of the primary outcomes.

Exclusion criteria were: (1) all study designs other than RCTs; (2) studies involving use of bioresorbable stents or bare metal stents; (3) studies comparing only IVUS-guided PCI with OCT-guided PCI and (4) studies that did not report the outcomes of interest. No language, sample size, duration of follow-up or date restrictions were applied.

### 2.3. Data Extraction

Two reviewers extracted data into an Excel spreadsheet by reviewing each article independently and resolving discrepancies through discussion with the third reviewer. Extracted data included study characteristics (including study design, country of origin, sample size and follow-up duration), baseline characteristics (including age, gender, hypertension, diabetes, prior percutaneous coronary intervention, prior myocardial infarction and other comorbidities) and outcomes of interest, including crude and risk estimates.

### 2.4. Risk of Bias and Quality Assessment

The quality of the studies included in the final analysis was assessed using the Cochrane Risk of Bias Tool for RCTs (RoB 2) [9]. Two authors evaluated each study across five domains: randomization and allocation, deviations from intended interventions, missing outcome data, measurement accuracy and selective reporting. Each study was then assigned a bias rating of low risk, some concerns or high risk based on this assessment.

### 2.5. Outcomes

We extracted data for the following clinical outcomes: (1) MACE, (2) TVF, (3) all-cause mortality, (4) cardiovascular mortality, (5) MI, (6) target vessel MI, (7) stent thrombosis, (8) target vessel revascularization (TVR), (9) target lesion revascularization (TLR) and (10) bleeding.

### 2.6. Statistical Analysis

We assessed outcomes on an intention-to-treat basis. Review Manager (RevMan 5.4.1) was used for statistical analysis of the outcomes. A random effects model was used to calculate the pooled risk ratio (RR) along with a 95% confidence interval (CI). I^2^ statistics was used to quantify unexplained statistical heterogeneity. Heterogeneity was classified as low, moderate or high on the basis of I^2^ values of 25%, 50% and 75%.

## 3. Results

### 3.1. Study and Patient Characteristics

**Figure 1:**
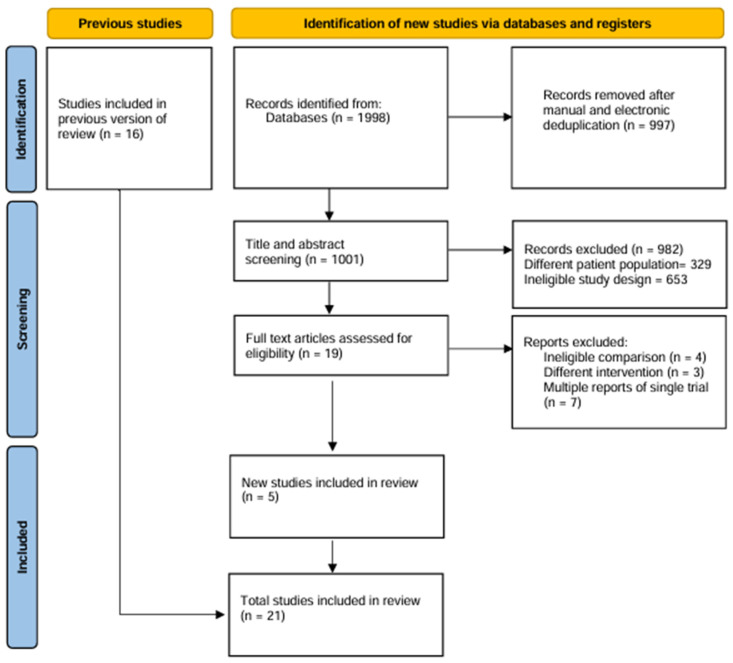
PRISMA flow diagram.

Following the primary and full text screening of retrieved articles, 21 RCTs [3,10,11,12,13,14,15,16,17,18,19,20,21,22,23,24,25,26,27,28,29] were finally included in our meta-analysis (Figure 1). These RCTs comprised of total of 18,043 patients who underwent PCI, 9415 allocated to imaging group and 8628 to angiography group. The follow-up duration across the studies varied from 6 months to 5 years. The age of the patients ranged from 57 to 75 years, with males comprising 73.6% of the cohort. Although visual inspection of funnel plots revealed asymmetry in some outcomes, formal assessment using Egger’s linear regression test did not indicate significant publication bias (Table S1). The characteristics of the included studies and patients are summarized in Table 1.

### 3.2. Quality Assessment of Included Studies

**Figure 2:**
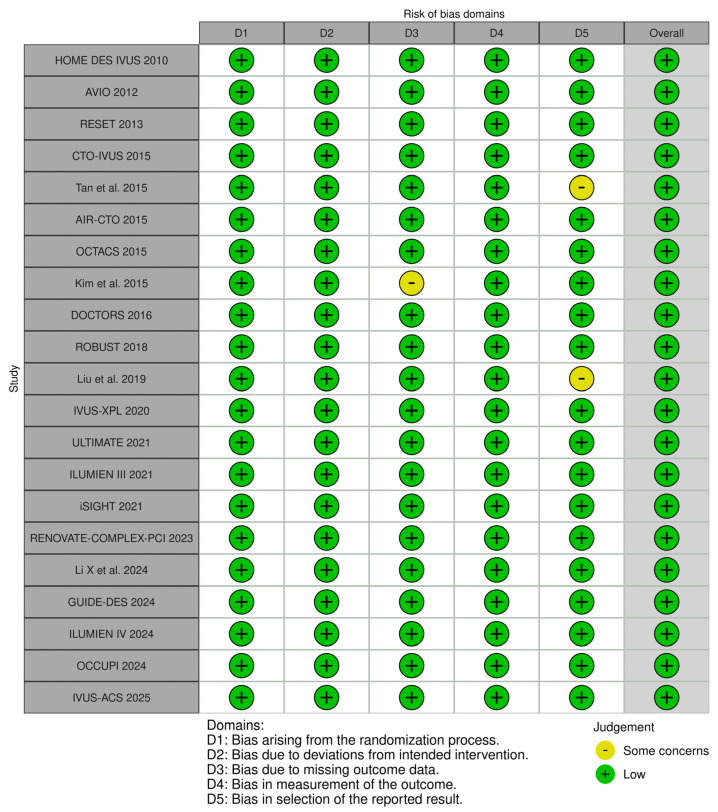
Quality assessment of the included studies. Chamie et al. (2021) [3], Gao et al. (2021) [10], Lee et al. (2023) [11], Hong et al. (2020) [12], Ali et al. (2021) [13], Antonsen et al. (2015) [14], Chieffo et al. (2013) [15], Jakabčin et al. (2010) [16], Kala et al. (2018) [17], Kim et al. (2015) [18], Kim et al. (2013) [19], Kim et al. (2015) [20], Meneveau et al. (2016) [21], Tan et al. (2015) [22], Tian et al. (2015) [23], Liu et al. (2018) [24], Lee et al. (2024) [25], Li et al. (2024) [26], Landmesser et al. (2024) [27], Hong et al. (2024) [28], Gao et al. (2025) [29].

The risk of bias assessment showed low risk across all 21 studies. However, some concerns were identified in two studies on a selection of reported results and in one study regarding missing outcome data (Figure 2).

### 3.3. Outcomes of Interest

#### 3.3.1. All-Cause Mortality

**Figure 3:**
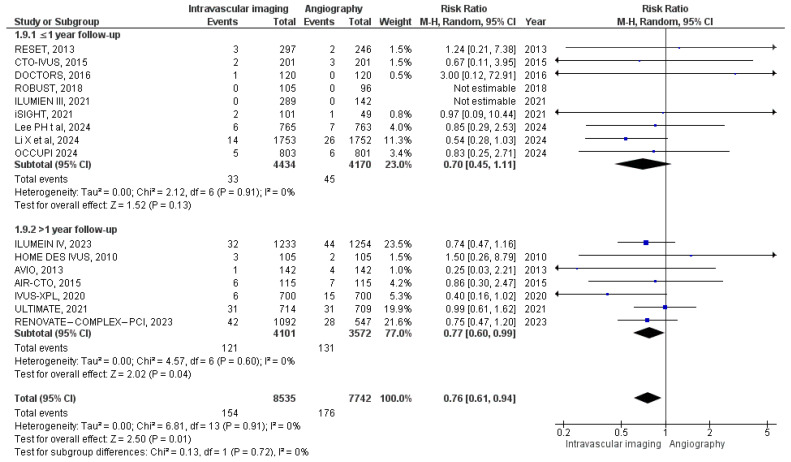
Forest plot of all-cause mortality. Chamie et al. (2021) [3], Gao et al. (2021) [10], Lee et al. (2023) [11], Hong et al. (2020) [12], Ali et al. (2021) [13], Chieffo et al. (2013) [15], Jakabcin et al. (2010) [16], Kala et al. (2018) [17], Kim et al. (2015) [18], Kim et al. (2013) [19], Meneveau et al. (2016) [21], Tian et al. (2015) [23], Lee et al. (2024) [25], Li et al. (2024) [26], Landmesser et al. (2023) [27], and Hong et al. (2024) [28].

All-cause mortality was reported in 16 studies. In the intravascular image-guided PCI group, there were 154 deaths among 8535 patients, compared to 176 deaths among 7742 patients in the angiography-guided PCI group. Meta-analysis yielded a RR of 0.76 (95% CI: 0.61 to 0.94; *p* = 0.01, I^2^ = 0%), indicating a statistically significant reduction in all-cause mortality with the intravascular imaging technique (Figure 3). Subgroup analysis based on follow-up duration showed a lower risk of all-cause mortality in the intravascular image-guided PCI group across both subgroups. However, the test for subgroup difference was not statistically significant (*p* = 0.72).

#### 3.3.2. Cardiac Mortality

**Figure 4:**
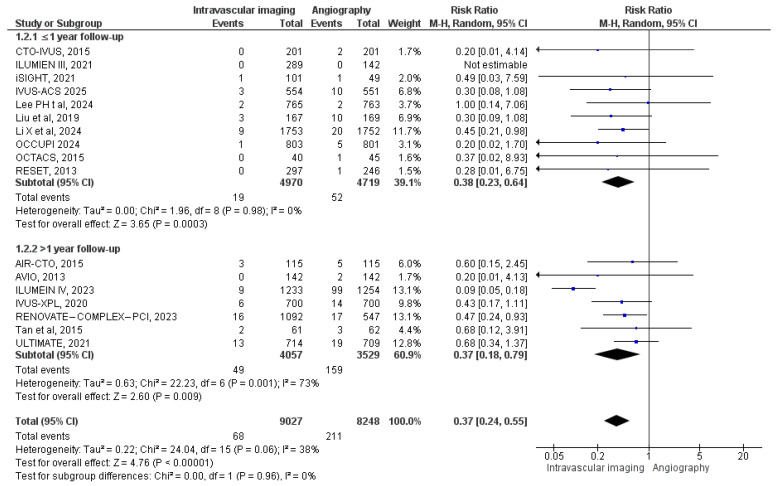
Forest plot of cardiac mortality. Chamie et al. (2021) [3], Gao et al. (2021) [10], Lee (2023) [11], Hong et al. (2020) [12], Ali et al. (2021) [13], Antonsen et al. (2015) [14], Chieffo et al. (2013) [15], Kim et al. (2015) [18], Kim et al. (2013) [19], Tan et al. (2015) [22], Tian et al. (2015) [23], Liu et al. (2018) [24], Lee et al. (2024) [25], Li et al. (2024) [26], Landmesser et al. (2023) [27], Hong et al. (2024) [28], and Gao et al. (2025) [29].

Cardiac mortality was reported in 17 studies. In the intravascular image-guided PCI group, 68 deaths were reported among 9027 patients, compared to 211 deaths among 8248 patients in the angiography-guided PCI group. Meta-analysis demonstrated a significantly lower risk of cardiac death with intravascular imaging guidance (RR: 0.37, 95% CI: 0.24 to 0.55; *p* < 0.00001, I^2^ = 38%) (Figure 4). Subgroup analysis based on follow-up duration showed a lower risk of cardiac mortality in the intravascular image-guided PCI group across both subgroups, with greater reduction observed in the >1-year subgroup (RR: 0.37). However, the test for subgroup difference was not statistically significant (*p* = 0.96).

#### 3.3.3. MACE

**Figure 5:**
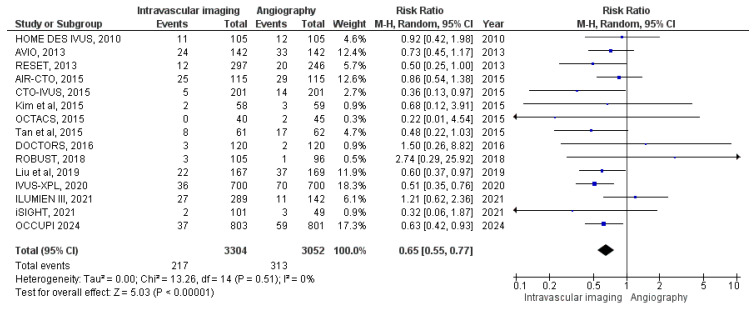
Forest plot of MACE. Chamie et al. (2021) [3], Hong et al. (2010) [12], Ali et al. (2021) [13], Antonsen et al. (2015) [14], Chieffo et al. (2013) [15], Jakabcin et al. (2010) [16], Kala et al. (2018) [17], Kim et al. (2015) [18], Kim, J.-S et al. (2013) [19], Kim et al. (2015) [20], Meneveau et al. (2016) [21], Tan et al. (2015) [22], Tian et al. (2015) [23], Liu et al. (2018) [24], Hong et al. (2024) [28].

MACE was reported in 15 studies. Among patients in the intravascular image-guided PCI group, 217 events occurred in a cohort of 3304 patients, while 313 events were observed among 3052 patients in the angiography-guided PCI group. The meta-analysis indicated a significantly lower risk of MACE in the intravascular imaging group (RR: 0.65, 95% CI: 0.55 to 0.77; *p* < 0.00001, I^2^ = 0%) (Figure 5).

#### 3.3.4. Target Vessel MI

**Figure 6:**
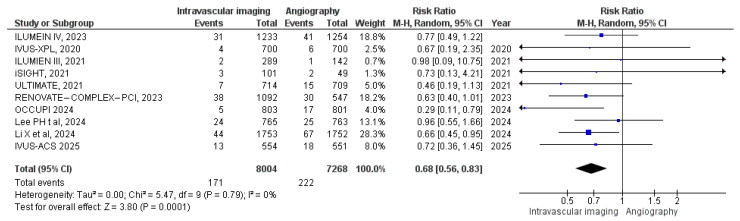
Forest plot of target vessel MI. Chamie et al. (2021) [3], Gao et al. (2021) [10], Lee et al. (2023) [11], Hong et al. (2010) [12], Ali et al. (2021) [13], Lee et al. (2024) [25], Li et al. (2024) [26], Landmesser et al. (2023) [27], Hong et al. (2024) [28], Gao et al. (2025) [29].

Ten studies reported Target Vessel MI. In the intravascular image-guided PCI cohort, there were 171 cases among 8004 patients, compared to 222 cases among 7268 patients in the angiography-guided group. Meta-analysis demonstrated a significantly reduced risk of target vessel MI with intravascular imaging (RR: 0.68, 95% CI: 0.56 to 0.83; *p* = 0.0001, I^2^ = 0%) compared with the angiography-guided technique (Figure 6).

#### 3.3.5. Stent Thrombosis

**Figure 7:**
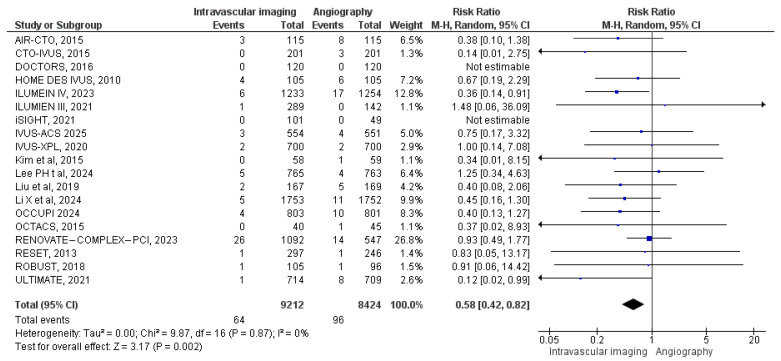
Forest plot of stent thrombosis. Chamie et al. (2021) [3], Gao et al. (2021) [10], Lee et al. (2023) [11], Hong et al. (2010) [12], Ali et al. (2021) [13], Antonsen et al. (2015) [14], Jakabcin et al. (2010) [16], Kala et al. (2018) [17], Kim et al. (2015) [18], Kim et al. (2013) [19], Kim et al. (2015) [20], Meneveau et al. (2016) [21], Tian et al. (2015) [23], Liu et al. (2018) [24], Lee et al. (2024) [25], Li et al. (2024) [26], Landmesser et al. (2023) [27], Hong et al. (2024) [28], Gao et al. (2025) [29].

Stent thrombosis was reported in 19 studies. A total of 64 cases were documented among 9212 patients in the intravascular image-guided PCI group, compared to 96 cases among 8424 patients in the angiography-guided PCI group. Meta-analysis revealed a significantly lower risk of stent thrombosis in the intravascular imaging group (RR: 0.58, 95% CI: 0.42 to 0.82; *p* = 0.002, I^2^ = 0%) (Figure 7).

#### 3.3.6. Target Vessel Revascularization

**Figure 8:**
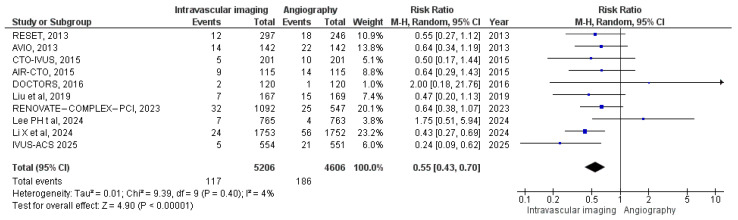
Forest plot of target vessel revascularization. Lee et al. (2023) [11], Chieffo et al. (2013) [15], Kim et al. (2015) [18], Kim et al. (2013) [19], Meneveau et al. (2016) [21], Tian et al. (2015) [23], Liu et al. (2018) [24], Lee et al. (2024) [25], Li et al. (2024) [26], and Gao et al. (2025) [29].

A total of 10 studies reported the rates of target vessel revascularizations. In the intravascular image-guided PCI group, there were 117 revascularizations among 5206 patients, compared to 186 revascularizations among 4606 patients in the angiography-guided group. Meta-analysis indicated a significantly lower risk of target vessel revascularization with intravascular imaging group (RR: 0.55, 95% CI: 0.43 to 0.70; *p* < 0.00001, I^2^ = 4%) compared with angiography-guided group (Figure 8).

#### 3.3.7. Target Lesion Revascularization

**Figure 9:**
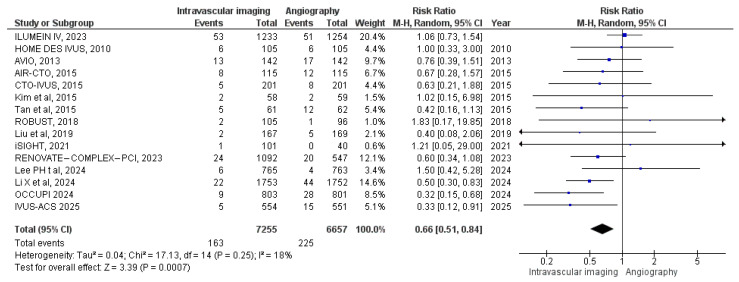
Forest plot of target lesion revascularization. Chamie et al. (2021) [3], Lee et al. (2023) [11], Chieffo et al. (2013) [15], Jakabcin et al. (2010) [16], Kala et al. (2018) [17], Kim et al. (2015) [18], Kim (2015) [20], Tan et al. (2015) [22], Tian et al. (2015) [23], Liu et al. (2018) [24], Lee et al. (2024) [25], Li et al. (2024) [26], Landmesser et al. (2023) [27], Hong et al. (2024) [28], Gao et al. (2025) [29].

Fifteen studies examined the occurrence of target lesion revascularization. In the intravascular image-guided PCI group, 163 revascularizations were recorded among 7255 patients, compared to 225 revascularizations among 6657 patients in the angiography-guided group. Meta-analysis showed a significantly reduced risk of target lesion revascularization with intravascular imaging guidance (RR: 0.66, 95% CI: 0.51 to 0.84; *p* = 0.0007, I^2^ = 18%) (Figure 9).

#### 3.3.8. Myocardial Infarction

**Figure 10:**
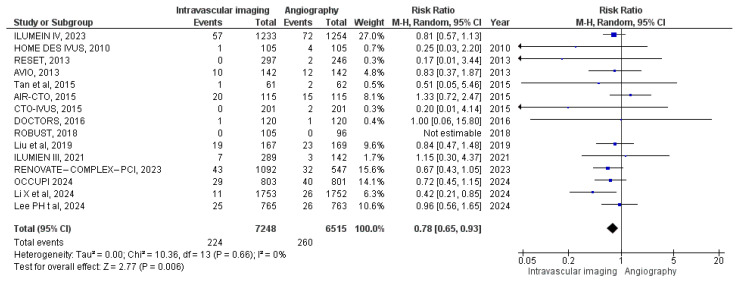
Forest plot of myocardial infarction. Lee et al. (2023) [11], Ali et al. (2021) [13], Chieffo et al. (2013) [15], Jakabčin et al. (2010) [16], Kala et al. (2018) [17], Kim et al. (2015) [18], Kim et al. (2013) [19], Meneveau et al. (2016) [21], Tan et al. (2015) [22], Tian et al. (2015) [23], Liu et al. (2018) [24], Lee et al. (2024) [25], Li et al. (2024) [26], Landmesser et al. (2024) [27], Hong et al. (2024) [28].

Fifteen studies provided data on the incidence of myocardial infarction. In the intravascular image-guided PCI group, there were 224 cases among 7248 patients, compared to 260 cases among 6515 patients in the angiography-guided group. Meta-analysis shows a statistically significant difference between the two groups (RR: 0.78, 95% CI: 0.65 to 0.93; *p* = 0.006, I^2^ = 0%) (Figure 10).

#### 3.3.9. Bleeding Events

**Figure 11:**
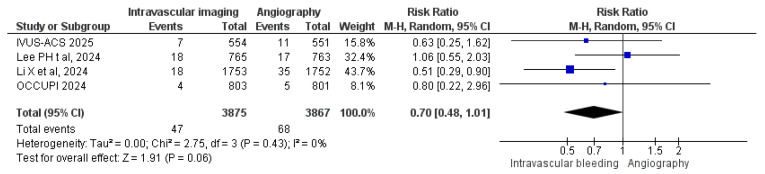
Forest plot of bleeding events. Lee et al. (2024) [25], Li et al. (2024) [26], Hong et al. (2024) [28], Gao et al. (2025) [29].

Four studies assessed bleeding complications. Among the intravascular image-guided PCI group, 47 cases of bleeding were observed among 3875 patients, compared to 68 cases among 3867 patients in the angiography-guided group. Meta-analysis revealed no statistically significant difference in bleeding risk between the two approaches (RR: 0.70, 95% CI: 0.48 to 1.01; *p* = 0.06; I^2^ = 0%) (Figure 11).

## 4. Discussion

Our study is the most recent systematic review and meta-analysis comparing IVI-guided PCI with angiography guided PCI. We analyzed a total of 21 randomized control trials amounting to a sample size of 18,043 patients. This analysis demonstrated the superiority of IVI-guided approach to PCI as compared to PCI alone, as reflected by improved clinical outcomes such as reductions in all-cause death, cardiac death, MACE, MI, target vessel MI, stent thrombosis, TVR and TLR by 24%, 63%, 35%, 22%, 32%, 42%, 45% and 34%, respectively. A key finding of our study is a significant reduction in all-cause death by using IVI-guided approach, a breakthrough not observed in previous studies. Furthermore, statistical heterogeneity was very low or zero across all the analyses signifying the consistency of the effects across all studies. We believe that the benefits of such amplitude should be sufficient to change the level of recommendation for IVI-guided PCI from class 2a to class 1.

The present study differs from the previously published meta-analysis one significant fronts [2,30,31,32,33]. Firstly, our analysis includes new trials which increases the sample size, thus increasing the power of our study by a significant margin. Moreover, the previous study showed an insignificant reduction in all cause death constrained by low statistical power. Our study, on the other hand, has shown a significant reduction in all cause death by 24% (*p* = 0.03) underscoring the effectiveness of IVI-guided approach. Our study extends the most elaborate insight into existing trial data in the field.

Complex coronary artery lesions are challenging and warrant a meticulous consideration of the optimum treatment strategy. Angiography has some disadvantages as it provides a two-dimensional view of a three-dimensional structure, lacking an in-depth comprehension of plaque characteristics and vessel size [34]. Different IVI modalities include IVUS and OCT, which are both used commonly. IVUS provides better tissue penetration and enables full thickness visualization which helps in making an optimum decision. On the other hand, OCT allows for better spatial resolution with high tissue characterization [3,35]. Nonetheless, both tools are useful, and their use depends on the individual’s specialty [36]. Furthermore, studies have also shown that IVUS is not superior to OCT [37]. The mechanism for improved outcomes by using IVI-guided PCI can be explained using multiple factors. IVI provides a high-resolution image along with extensive information about plaque characteristics and vessel size [38]. It allows for optimal stent placement while avoiding malposition and helps detect edge dissection that might not be visualized with coronary angiography [39,40]. Additionally, IVI can also augment the safety and effectiveness of atherectomy procedures for calcified lesions [41,42]. Furthermore, IVI might potentially be linked with a reduced risk of periprocedural MI, which is a seen with PCI and is associated with worse prognosis [43]. This reduced risk of periprocedural MI might contribute to the decreased all-cause mortality associated with IVI-guided PCI.

To the best of our knowledge, no previous meta-analyses have demonstrated a significant reduction in all-cause death and myocardial infarction associated with IVI-guided PCI. While a meta-analysis by Buccheri S et al. did find a significant decrease in all cause death, this may be attributed to the observational studies included in that analysis [44]. Our meta-analysis, consisting solely of RCTs, is the first of its kind in the literature to suggest a significant reduction in all-cause death. A reduction in MI, TVR and TVL are good prognostic indicators, which could explain the decrease in all-cause death following IVI-guided PCI. Regardless of the mechanism, this breakthrough finding holds the potential to reshape clinical guideline procedures for PCI.

Despite the robust evidence supporting the superior nature of IVI over angiography guided PCI, the use of IVI-guided approach is not prevalent. According to a nationwide U.S. study, the use of intravascular imaging was less than 5% between 2004 and 2014 [6]. Some reasons for this might be lack of information about the imaging equipment, reservations about increase in procedural time and high costs and gap in knowledge about the images. However, the clinical advantages are that IVI comes with little or no downside as there is only a minute cost of imaging catheter and an insignificant increase in procedural lapse. On the contrary, an IVI-guided approach has shown to be cost effective as it alleviates the burden on the healthcare system by preventing hospitalizations and urgent TVR [45]. This further elucidates a stringent need for promoting IVI-guided approaches amongst clinicians. Interventional cardiologists should advocate for the use of IVI-guided techniques and should push for guideline upgradation. Moreover, IVI should no longer be considered an option for PCI, as it is superior to angiography in terms of various outcomes as shown consistently by contemporary evidence. This will not lead to improved outcomes among patients undergoing PCI but also enhance patient care.

With the addition of new trials, our study was able to highlight a significant decrease in all cause death by using an IVI-guided approach as compared to angiography alone. This is a hallmark finding of our study that makes it distinct from previous analyses. Along with this, the IVI-guided approach also reduced MACE, cardiac death, TVR and stent thrombosis [46]. Our findings advocate the use of IVI as standard care for PCI patients, also necessitating the revision of contemporary guidelines to align with the compelling evidence from randomized trials. Moreover, efforts should be directed towards identifying and overcoming the barriers behind the skepticism towards the IVI-guided approach.

## 5. Strengths and Limitations

Our study has various strengths owing to the large sample size, thus enhancing statistical power to elucidate clinically significant reductions in all-cause death, MACE and cardiac death with IVI-guided PCI. Moreover, our findings were consistent across trials as indicated by the low heterogeneity, further strengthening the reliability of our results. However, there are a few limitations that must be acknowledged. Firstly, we did not use individual patient data and only utilized published data, a limitation that is common to most meta-analyses. However, our thorough search and screening strategy yielded relevant studies from all major medical databases. Secondly, the outcomes definitions and reporting lacked consistency across trials. This challenge would be difficult to tackle even with the availability of individual patient data as it would necessitate an independent retrospective adjudication of events. Thirdly, we did not analyze the effects of IVUS and OCT separately, which could obscure the individual benefits of each modality. Nevertheless, our analysis is the most rigorous and comprehensive synthesis of current evidence, signifying the superiority of IVI-guided PCI over angiography-guided PCI.

## 6. Conclusions

In conclusion, IVI is associated with a significant reduction in all-cause mortality, MACE, cardiac death, MI, stent thrombosis, TVR and TLR in individuals undergoing PCI. The consistency of these findings across contemporary randomized evidence underpin the clinical utility of IVI for patients undergoing PCI. While this study-level meta-analysis shows promise for IVI-guided PCI, further patient level analyses will help strengthen the role of IVI-guided PCI and thus inform future guideline considerations.

## Figures and Tables

**Table 1:** Baseline characteristics of the included studies.

Trial	Year	Design	Region	Study Period	Follow-Up	Arms	*N*	Age (Years)	Male	HTN *n* (%)	DLD *n* (%)	Diabetes *n* (%)	Current Smoker *n* (%)	CHF	LVEF (%)	Prior MI *n* (%)	Prior PCI *n* (%)	Prior CABG *n* (%)	SA *n* (%)	UA *n* (%)	STEMI/Acute MI *n* (%)	UA/NSTEMI *n* (%)
HOME DES IVUS [16]	2009	Prospective, single-center RCT	Czech Republic	Jan 2004–Dec 2005	18 mo	AG	105	60.2 ± 11	75 (71)	75 (71)	69 (66)	47 (45)	37 (35)			34 (32%)	15 (14)	11 (10)	42 (40)		22 (21)	41 (39)
						IVUS	105	59.4 ± 13	77 (73)	70 (67)	66 (63)	44 (42)	42 (40)			39 (37%)	18 (17)	15 (14)	40 (38)		31 (29)	45 (43)
AVIO [15]	2012	Multicenter, open-label, investigator- driven RCT	International	May 2008–July 2011	2 y	AG	142	63.6 ± 11.0	109 (77)	95 (66.9)	109 (76.8)	38 (26.8)	44 (31.0)		55.9 ± 8.6					37 (26.1)		
						IVUS	142	63.9 ± 10.1	117 (82)	100 (70.4)	100 (70.4)	34 (23.9)	49 (34.5)		55.3 ± 8.5					42 (29.6)		
RESET Substudy [19]	2013	Prospective, open-label, multicenter RCT	South Korea	Apr 2009–Dec 2010	1 y	AG	269	64.5 ± 8.6	130 (52.8)	156 (63.4)	144 (58.5)	77 (31.3)	38 (15.4)		53.9 ± 25.1	8 (2.9)			133 (54.1)	92 (37.4)	21 (8.5)	
						IVUS	274	62.8 ± 9.2	197(66.3)	187 (63.0)	190 (64.0)	90 (30.3)	67 (22.6)		55.2 ± 23.9	3 (1.1)			151 (50.8)	116 (39.1)	30 (10.1)	
OCT-ACS [14]	2015	Prospective, single-center RCT	Denmark	Aug 2011–May 2013	6 mo	AG	45	62.6 ± 11.0	34 (68)	28 (56.0)		5 (10.0)	18 (36.0)			0	2 (4.0)					
						OCT	40	61.8 ± 9.4	36 (72)	28 (56.0)		8 (16.0)	23 (46.0)			2 (4)	3 (6.0)					
Kim et al. [20]	2015	Prospective, single-center, open-label RCT	South Korea	Dec 2011–Dec 2012	1 y	AG	59	61.6 (9.7)	37 (72.5)	25 (49.0)	37 (72.5)	16 (31.4)	15 (29.4)		63.6 (8.6)	8 (2)			31 (60.8)		20 (39.2)	
						OCT	58	58.8 (10.8)	39 (78)	27 (54.0)	33 (66.0)	16 (32.0)	16 (32.0)		64.2 (7.4)	3 (6)			31 (62)		19 (38.0)	
CTO-IVUS [18]	2015	Prospective, multicenter RCT	South Korea	Mar 2012–Aug 2013	1 y	AG	201	61.4 ± 10.1	162 (80.6)	128 (63.7)		68 (33.8)	69 (34.3)	10 (5)	56.7 ± 11.4	16 (8)	32 (15.9)	5 (2.5)				
						IVUS	201	61.0 ± 11.1	162 (80.6)	126 (62.7)		70 (34.8)	71 (35.3)	12 (6)	56.9 ± 13.1	16 (8)	31 (15.4)	3 (1.5)				
Tan et al. [22]	2015	Single- center, open- label RCT	China	Oct 2009–Sep 2012	2 y	AG	62	75.85 ± 3.49	43 (70)	29 (46.8)		18 (29.5)	29 (46.8)		53.33 ± 7.14	13 (21)			21 (34)	41 (66)		
						IVUS	61	76.54 ± 4.95	38 (62)	25 (41.0)		21 (34.4)	27 (44.3)		55.32 ± 5.02	10 (16.4)			18 (30)	43 (71)		
AIR-CTO [23]	2015	Multicenter RCT	China	Oct 2010–Nov 2011	2 y	AG	115	66 ± 11	92 (80)	81 (70.4)	32 (27.8)	31 (27.0)	45 (39.1)			35 (30.4)	24 (20.9)	5 (4.3)	87 (75.7)	11 (9.6)	17 (14.8)	
						IVUS	115	67 ± 10	102 (88.7)	86 (74.8)	25 (21.9)	34 (29.6)	45 (39.1)			24 (20.9)	23 (20)	3 (2.6)	82 (71.3)	10 (8.7)	23 (20.0)	
DOCTORS [21]	2016	Prospective, multicenter RCT	France	Sep 2013–Dec 2015	6 mo	AG	120	60.2 ± 11.3	91 (75.8)	50 (41.7)	56 (46.7)	19 (15.8)	51 (42.5)							9 (7.5)		
						OCT	120	60.8 ± 11.5	95 (75.2)	67 (55.8)	59 (49.2)	26 (21.7)	47 (39.2)							10 (8.3)		
ROBUST Substudy [17]	2017	Multicenter, open-label RCT	Czech Republic	Feb 2011–Oct 2012	9 mo	AG	96	59 (47–72)	84 (87)	50 (52)		25 (26)	57 (59)			6 (6)	3 (4)					
						OCT	105	57 (46–70)	87 (83)	53 (50)		18 (17)	67 (64)			1 (1)	4 (4)					
Liu et al. [24]	2019	Open-label, single-blind RCT	China	Dec 2010–Dec 2015	1 y	AG	169	64.9 ± 11.2	108 (63.9)	122 (72.2)	64 (37.9)	52 (30.8)	60 (35.5)	33 (19.2)	58.4 ± 10.5	24 (14.2)	28 (16.6)	2 (1.2)	18 (10.7)	126 (74.6)	21 (12.4)	
						IVUS	167	65.3 ± 10.6	106 (63.5)	116 (69.5)	63 (37.7)	56 (33.5)	62 (37.1)	31 (18.6)	55.6 ± 11.7	29 (17.4)	33 (19.8)	2 (1.2)	20 (12.0)	127 (76)	17 (10.2)	
IVUS-XPL [12]	2020	Investigator- initiated, multicenter RCT	South Korea	Oct 2010–Jul 2014	5 y	AG	700	63 ± 9	409 (69)	373 (63)	458 (65)	223 (38)	134 (23)		62.3 ± 10.2	27 (5)	60 (10)	16 (3)	307 (52)	189 (32)	98 (17)	
						IVUS	700	63 ± 9	408 (69)	382 (65)	471 (67)	189 (32)	155 (22)		62.8 ± 9.8	30 (5)	66 (11)	16 (3)	291 (49)	211 (36)	87 (15)	
ULTIMATE [10]	2021	Prospective, multicenter, investigator- initiated RCT	China	Aug 2014–Oct 2020	3 y	AG	709	65.9 ± 9.8	530 (73.2)	521 (72.0)	400 (55.2)	226 (31.2)									567 (78.3)	
						IVUS	714	65.2 ± 10.9	535 (73.9)	512 (70.7)	389 (53.7)	217 (30.0)									569 (78.6)	
ILUMIEN III: OPTIMIZE PCI [13]	2021	Prospective, 3-arm, single-blind, multicenter RCT	29 International centers	May 2015–Apr 2016	1 y	AG	142	67 (56–75)	104 (73)	107 (75)	109 (77)	40 (28)	33 (23)			32 (22)	15 (10)	8 (5)	50 (35)		51 (36)	
						IVUS	136	66 (61–73)	101 (74)	106 (78)	102 (75)	49 (36)	18 (13)			29 (20)	8 (5)	11 (8)	48 (35)		49 (36)	
						OCT	153	66 (59–72)	106 (69)	119 (78)	112 (73)	50 (33)	26 (17)			35 (22)	11 (7)	3 (2)	52 (34)		50 (33)	
iSIGHT [3]	2021	Prospective, single-center, active-controlled, noninferiority RCT	Brazil	Jan 2015–Dec 2016	1 y	AG	49	58.59 ± 10.2	38 (77.5)	39 (79.6)	28 (57.2)	22 (44.9)	14 (28.6)			17 (34.7)	14 (28.6)		21 (42.9)	16 (32.6)	12 (24.5)	
						IVUS	50	59.32 ± 10.37	36 (72)	42 (84)	30 (60)	20 (40)	14 (28)			17 (34)	13 (26)		18 (36)	22 (44)	10 (20.0)	
						OCT	51	59.92 ± 8.92	31 (60.8)	46 (90.2)	36 (70.6)	17 (33.3)	17 (33.3)			15 (29.4)	12 (23.5)		22 (43.1)	20 (39.2)	9 (17.7)	
RENOVATE- COMPLEX- PCI [11]	2023	Prospective, multicenter, investigator-initiated, open-label, RCT	South Korea	2020–2021	2.1 y	AG	547	66.0 ± 10.0	431 (78.8)	323 (59.0)	280 (51.2)	246 (45)	95 (17.4)		59.3 ± 11.0	42 (7.7)	127 (23.2)		275 (50.3)	173 (31.6)	111 (20)	87 (15.9)
						IVUS/OCT	1092	65.3 ± 10.3	869 (79.6)	682 (62.5)	560 (51.3)	422 (39)	212 (19.4)		58.4 ± 11.9	75 (6.9)	268 (24.5)		532 (48.7)	361 (33.1)	227 (21)	171 (15.7)
Lee, PH et al. [25]	2024	Open-label, multicenter, noninferiority, RCT	Korea	Feb 2017–Aug 2021	1 y	AG	763	64.1 (9.9)	574 (75.2)	480 (62.9)	655 (85.8)	257 (33.7)				47 (6.2)	117 (15.3)	7 (0.9)			53 (6.9)	166 (21.8)
						IVUS	765	64.6 (9.5)	622 (81.3)	488 (63.8)	649 (84.8)	237 (31.0)				56 (7.3)	119 (15.6)	6 (0.8)			56 (7.3)	171 (22.4)
Li X et al. [26]	2024	Two-stage, multicentre, RCT	China, Italy, Paksitan and UK	Aug 2019–Oct 2022	1 y	AG	1752	63 (54–69)	1299 (74.1)	1089 (62.2)	1222 (69.8)	551 (31.5)	487 (27.8)	106 (6.1)	62 (55–65)	154 (8.8)	179 (10.2)	4 (0.2)		726 (41.4)	489 (27.9)	537 (30.7)
						IVUS	1753	62 (54–69)	1285 (73.3)	1103 (62.9)	1187 (67.7)	554 (31.6)	499 (28.5)	111 (6.3)	62 (55–65)	152 (8.7)	179 (10.2)	4 (0.2)		699 (39.9)	484 (27.6)	570 (32.5)
OCCUPI [28]	2024	Open-label, multicentre, RCT	South Korea	Jan 2019–Sept 2022	1 y	AG	801	64 (58–70)	644 (80)	451 (56)	661 (83)	262 (33)	158 (20)		59.7 (10.1)	42 (5)	159 (20)	14 (2)	423 (53)	215 (27)	58 (7)	105 (13)
						OCT	803	64 (57–70)	646 (80)	466 (58)	684 (85)	261 (33)	149 (19)		59.5 (8.8)	40 (5)	171 (21)	10 (1)	391 (49)	248 (31)	46 (6)	118 (15)
ILUMIEN IV [27]	2024	Prospective, single blind, RCT	North America, Europe, Middle East, and Asia-Pacific region	May 2018–Dec 2020	2 y	AG	1072	65.4 ± 10.4	810 (75.6)	784 (73.1)	727 (67.8)	436 (40.7)	216/1071 (20.2)		55.3 ± 8.6	256 (23.9)	135/1060 (12.7)	41 (3.8)	314 (29.3)	288 (26.9)	63 (5.9)	239 (22.3)
						OCT	1056	65.2 ± 10.5	827 (78.3)	743 (70.4)	684 (64.8)	440 (41.7)	211 (20.0)		55.2 ± 8.5	216 (20.5)	134/1035 (12.9)	49 (4.6)	296 (28.0)	291 (27.6)	59 (5.6)	265 (25.1)
IVUS-ACS [29]	2025	RCT	China, Italy, Pakistan, UK	Aug 2019–Oct 2022	1 y	AG	551	62 ± 10	384 (69.7)	403 (73.1)	429 (77.9)	145 (26.3)	135 (24.5)		58 ± 10	58 (10.5)	67 (12.2)	2 (0.4)		212 (38.5)	171 (31.0)	168 (30.5)
						IVUS	554	63 ± 10	372 (67.1)	391 (70.6)	395 (71.3)	148 (26.7)	141 (25.5)		58 ± 9	63 (11.4)	64 (11.6)	0 (0)		217 (39.2)	143 (25.8)	194 (35.0)

(OCT, optical coherence tomography; IVUS, Intravascular ultrasound guided; AG, angiography guided; HTN, Hypertension; CHF, Congestive heart failure; LVEF, left ventricular ejection fraction; MI, Myocardial infarction; PCI, percutaneous coronary intervention; CABG, coronary bypass grafting; SA, stable angina; UA, unstable angina; STEMI, ST-elevation myocardial infarction, NSTEMI; Non ST-elevation myocardial infarction).

## Data Availability

Publicly available datasets were analyzed in this study. This data can be found here: [https://pubmed.ncbi.nlm.nih.gov/].

## Supplementary Materials

The following supporting information can be downloaded at: https://www.mdpi.com/article/10.3390/diagnostics15091175/s1, Table S1: Summary of Egger’s Test conducted for each outcome.
