# Supplementary material for: Intravascular Imaging-Guided Versus Angiography-Guided Percutaneous Coronary Intervention: A Systematic Review and Meta-Analysis of Randomized Controlled Trials
Source: Diagnostics (Basel). 2025 May 6;15(9):1175. doi: 10.3390/diagnostics15091175 (PMC12071307; doi:10.3390/diagnostics15091175)
Supplement: Supplementary file 1 [file diagnostics-15-01175-s001.zip › diagnostics-3547346-supplementary.pdf]

Supplementary:

Table S1: Summary of Egger's Test conducted for each outcome

**Regression Test for Funnel Plot Asymmetry**								
	**All-cause mortality**	**Cardiac mortality**	**MACE**	**Target vessel MI**	**Stent thrombosis**	**Target vessel revasculari zation**	**Target lesion revasculari zation**	**MI**
**Parameter**	**Value**							
Model	weighted regression with multiplicative dispersion							
Predictor	standard error							
Test for funnel plot symmetry								
Test Statistic (t)	0.264	0.3238	0.2717	-0.6256	-1.0461	1.0514	0.7179	-1.2118
Degrees of Freedom (df)	12	14	13	8	15	8	13	12
p-value	0.7962	0.7509	0.7901	0.549	0.3121	0.3238	0.719	0.2489
Limit Estimate (as sei -> 0)								
b	-0.3128	-1.1626	-0.4687	-0.2854	-0.2639	-0.9309	-0.3013	-0.1011
95% CI Lower Bound	-0.6564	-1.9519	-0.8512	-0.7024	-0.8871	-1.7017	-0.8333	-0.4169
95% CI Upper Bound	0.0307	-0.3732	-0.0861	0.1316	0.3594	-0.1602	0.2308	0.2147
